# Investigation of TiO_2_ Deposit on SiO_2_ Films: Synthesis, Characterization, and Efficiency for the Photocatalytic Discoloration of Methylene Blue in Aqueous Solution

**DOI:** 10.3390/nano13081403

**Published:** 2023-04-19

**Authors:** Yuliana de Jesús Acosta-Silva, Manuel Toledano-Ayala, Salvador Gallardo-Hernández, Luis A. Godínez, Arturo Méndez-López

**Affiliations:** 1 Research and Postgraduate Division, Faculty of Engineering, Autonomous University of Queretaro (UAQ), University Center, Querétaro 76010, Mexico; 2 Department of Physics, Center for Research and Advanced Studies of the National Polytechnic Institute, México City 07360, Mexico; 3 Centro de Investigación en Química para la Economía Circular, CIQEC, Faculty of Chemistry, Autonomous University of Queretaro, University Center, Querétaro 76010, Mexico

**Keywords:** dip-coating, TiO_2_-SiO_2_ thin films, photocatalytic processes, methylene blue, sol-gel, UV-visible light

## Abstract

TiO_2_-SiO_2_ thin films were created on Corning glass substrates using a simple method. Nine layers of SiO_2_ were deposited; later, several layers of TiO_2_ were deposited, and their influence was studied. Raman spectroscopy, high resolution transmission electron spectroscopy (HRTEM), an X-ray diffractometer (XRD), ultraviolet-visible spectroscopy (UV-Vis), a scanning electron microscope (SEM), and atomic force microscopy (AFM) were used to describe the sample’s shape, size, composition, and optical characteristics. Photocatalysis was realized through an experiment involving the deterioration of methylene blue (MB) solution exposed to UV-Vis radiation. With the increase of TiO_2_ layers, the photocatalytic activity (PA) of the thin films showed an increasing trend, and the maximum degradation efficiency of MB by TiO_2_-SiO_2_ was 98%, which was significantly higher than that obtained by SiO_2_ thin films. It was found that an anatase structure was formed at a calcination temperature of 550 °C; phases of brookite or rutile were not observed. Each nanoparticle’s size was 13–18 nm. Due to photo-excitation occurring in both the SiO_2_ and the TiO_2_, deep UV light (λ = 232 nm) had to be used as a light source to increase photocatalytic activity.

## 1. Introduction

Diverse colors and other organic and inorganic contaminants are found in textile industry wastewater. Twelve percent of synthetic textile dyes, including eriochorome black-T (EBT), methyl orange (MO), rhodamine B, and methylene blue (MB), are thought to be lost during the dyeing process, and approximately 20% of these dyes are carried as waste to industrial wastewater treatment facilities [1]. Among these chemicals, MB is one of the most popular, as it is used not only as a dye for wool, hair coloring compounds, paper prints, and cotton, but also as an antiseptic, among other health-related purposes [2]. MB, on the other hand, is regarded as biologically dangerous, because it is a powerful carcinogen for marine animals, and in humans severely irritates the eyes, causes convulsions, irritates sensitive skin, and induces tachycardia [3]. MB also reacts with various substances, which makes the treatment of aqueous effluents containing MB a difficult task [4]. On the other hand, advanced oxidation processes (AOPs) are regarded as efficient approaches for the elimination of organic contaminants from aqueous solutions, such as MB. These procedures are based on the fact that hydroxyl radicals (HO**•**), which have a particularly high oxidation potential, rapidly oxidize the majority of organic contaminants with great efficiency (generally in the range 108–1011 M^−1^ s^−1^) [5,6,7]. One way to produce OH radicals is by the oxidation of surface adsorbed H_2_O molecules, using photo-excited semiconductor materials [8]. Titanium dioxide (TiO_2_) is an excellent photocatalyst, due to its low cost, nontoxicity, and good chemical and mechanical stability [9,10]. Therefore, TiO_2_ is used in solar cells [11], self-cleaning glasses [12], antifogging windows [13], and as a photocatalytic material for the degradation of contaminants in wastewater [14]. The ability of illuminated TiO_2_ to produce OH radicals depends on specific features of TiO_2_, such as the size of the semiconductor crystallites and their surface area [15]. The photo-physical and chemical properties of the TiO_2_ material allows for coupling to other semiconductors; in this regard, SiO_2_ is a practical option because of its great mechanical strength and good thermal stability [16]. Amorphous SiO_2_ has also been demonstrated to have a high surface area and outstanding adsorption capacity, making it an effective substrate for semiconductor films [17]. Zhou et al., for example, demonstrated that mixed metal oxides (TiO_2_-SiO_2_) improve the photocatalytic efficiency of the individual materials, because it enhances the materials’ adsorption properties and increases their concentration of surface hydroxyl groups in the thin film. In addition, SiO_2_ was shown to promote a large surface area and an appropriately porous structure [15]. There are abundant reports in the literature focusing on the synthesis and characterization of TiO_2_-based photocatalysts; in this context, we believe that in addition to phase structure, doping, and composite design and synthesis, the study of TiO_2_ layers grown by the sol–gel dip-coating method on top of the SiO_2_ semiconductor material films could be useful for the development of novel and efficient photocatalytic surfaces. This communication, therefore, presents the structural, morphological, and optical properties of the SiO_2_/TiO_2_ composite films proposed, as well as an exploration of their photocatalytic performance.

## 2. Experimental Details

### 2.1. SiO_2_ Thin Films

**Figure 1:**
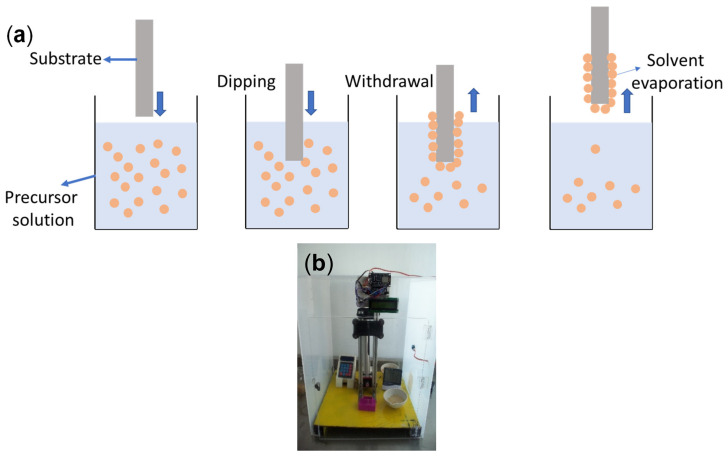
(**a**) Sequential steps of the sol–gel dip-coating method for thin film deposition. (**b**) Dip coating system assembled in the NanoBiotechnology and Photocatalysis Laboratory—UAQ. This system is used for the production of various thin films.

SiO_2_ solutions were prepared by dissolving a certain amount of triblock copolymer (BASF, EO106-PO70-EO106, F127) in ethanol (J.T. Baker, Querétaro, Mexico), with constant stirring for 1 h at 37 °C. Then, 0.7 mL of HCl (J.T. Baker) were added by drops, and the solution was continuously stirred for 30 min. After adding 5.5 mL of TEOS to the mixture, it was agitated for 24 h at 36 °C. The resulting transparent and homogeneous phase was then employed to prepare thin films on glass substrates (Corning 2947, area 2.5 × 7.5 cm^2^) using the dip-coating technique. Figure 1a shows a scheme describing the dip-coating process employed in this work. The previously cleaned substrate is dipped into a solution of the precursors (the material to be deposited) and then withdrawn vertically at a controlled speed. For this purpose, the homemade system shown in Figure 1b (operating at a withdrawal rate of 8 cm/min) was utilized to obtain SiO_2_ thin films containing nine sequential coatings; each layer was prepared by drying the coating at 250 °C for 2 h in an open atmosphere. Subsequently, the temperature was increased to 550 °C (calcination temperature) over two hours at a rate of 2 °C/min ramp.

### 2.2. TiO_2_-SiO_2_ Thin Films

Thin films were prepared by immersion as reported in a previous section, in a solution containing titanium (IV) isopropoxide (Sigma-Aldrich Co., Querétaro, Mexico), 2-propanol (J.T. Baker), and hydrochloric acid (37% HCl). The TiO_2_ thin films were deposited using the 2 cm/min removal rate for the dip coating process, and in this case, the films surveyed consisted of coatings of 5, 7, and 9 layers. As was indicated for the SiO_2_ films, each layer was dried at 250 °C for 3 min, and once each film was completed with the corresponding number of layers, the surface modified material was calcined at 550 °C for 1 h. In terms of the cross-sectional characterization of the composite films under study, we carried out thickness measurements using a KLA TENCO P-15 profilometer as described in the experimental section. In this way, while the thickness of the SiO_2_ films under study corresponded to ~250 nm, the thicknesses of the TiO_2_ material films consisted of ~171, 219, and 262 nm, depending on the number of deposited TiO_2_ layers (5, 7 and 9, respectively).

### 2.3. Structural, Optical, and Morphological Characterization of the Films

UV-Vis measurements were carried out using an Evolution 220 UV-Vis Spectrophotometer. X-ray diffraction experiments were carried out using a Philips X-ray diffractometer (PANalytical’s X’pert PRO X-ray diffractometer, Malvern, UK) that employs a Cu-Kα radiation with a wavelength of 0.15405 nm in the 20 ≤ 2θ ≤ 80° range. The voltage and current settings were 30 kV and 40 mA, respectively. The samples were continuously scanned with a step size of 0.02° (2θ) and a count time of 1 s per step. Structural properties were also studied using Raman spectroscopy that collected data using a Labram-Dilor Raman spectrometer equipped with a He-Ne laser exciting source, operating between the wavelengths of 200 and 800 nm at ambient temperature (AT). The roughness and surface topography were examined by AFM (Park Scientific Inst. System, Suwon, South Korea). Using a scanning electron microscope (SEM, JEOL JSM-6300, Tokyo, Japan), surface pictures were acquired. Prior to the acquisition of images by high-resolution transmission electron microscopy, tiny bits of films were scraped. JEOL JEM 2010 microscope with an acceleration voltage of 200 kV was used for this objective. Typically, the magnification ranged from ×400,000 to ×500,000. At the camera length L = 20 cm, selected area electron diffraction (SAED) was carried out. Thickness measurements were made using a KLA TENCOR P-15 profilometer (Milpitas, CA, USA).

### 2.4. Photocatalytic Activity Evaluation

The PA of films was determined at AT by assessing the MB discoloration kinetics of aqueous solutions of 5.4 mg/L (1.88 × 10^−5^ mol/L) under UV radiation. In this way, 3 mL of the aqueous MB solution were poured into a standard quartz cell, and later, a TiO_2_-SiO_2_ coated substrate was placed vertically inside the cell (with a 2 cm^2^ exposed area). Five quartz cells prepared in this way were used for each thin film under study. The 15 W lamp used to produce the irradiation light had a 254 nm wavelength (G15T8 germicidal lamp as the exciting source). Five centimeters separated the quartz cells from the lamp. A Thermo Scientific^TM^ Evolution 220 Spectrophotometer was used to measure the optical absorbance of the test solution every 60 min for the course of the 5 h of total irradiation. The absorbance peak reduction for MB was examined in the range of 400 to 800 nm. The residual methylene blue concentration was calculated using the absorbance data at 664 nm employing the Beer-Lambert’s law.

## 3. Results and Discussion

### 3.1. X-ray Diffraction

**Figure 2:**
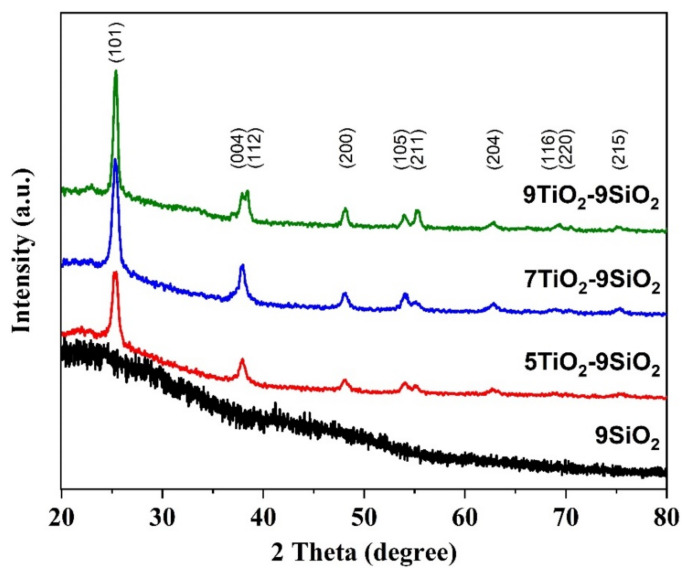
X-ray diffraction patterns for samples of TiO_2_ over SiO_2_ annealed. The tetragonal phase is linked to thin films of TiO_2_ that correspond to the (hkl) indices.

The XRD patterns of the thin films of TiO_2_ deposited on mesoporous SiO_2_ thin films are shown in Figure 2. The JCPDS-ICDD powder diffraction database was used to identify the crystal phases and matching miller indices. The JCPDF number for TiO_2_ is 00-21-1272. The anatase phase of TiO_2_ predominated in all the samples’ patterns; no other phase was present. The samples diffraction peaks were seen at 2θ values of 25.3°, 37.8°, 38.6°, 48.1°, 53.9°, 55.1°, 62.7°, 68.9°, 70.4°, and 75.1° and could be perfectly correlated with the (101), (004), (112), (200), (105), (211), (204), (116), (220), and (215) crystal planes of anatase TiO_2_. All peaks were well indexed according to the standard patterns. The increase in the number of TiO_2_ coatings resulted in thin films that exhibited higher intensities, indicating an increase in crystallinity owing to the higher presence of TiO_2_ layers on top of SiO_2_. No phase transformation occurred for any of the samples. Moreover, all samples displayed a preferential orientation growth at (101) the plane (see Figure 2), which can be attributed to the plane’s low surface energy value [18]. According to Zhang et al. [19], the phase transformation of TiO_2_ only takes place at temperatures above 900 °C. An estimation of average crystallite size was calculated using the Scherrer equation; 5TiO_2_-9SiO_2_, 7TiO_2_-9SiO_2_, and 9TiO_2_-9SiO_2_ samples correspond to 13, 14, and 18 nm, respectively. For these calculations, Equation (1) was used as follows [20]:

(1) $$d=\frac{K\lambda }{\beta \cos\theta }\;$$

*λ* the wavelength of the radiation (1.54056 for CuK radiation), *θ* is Bragg’s angle, *β* the full width at half maximum (FWHM) intensity in radians, d the average thickness of the crystal in a direction normal to the diffracting plane (hkl), and *K* is Scherrer constant. Equations (2) and (3), respectively, express two additional structural parameters known as the dislocation density (*δ*) and the microstrain (*ε*) [21]:

(2) $$\delta =\frac{1}{d^{2}}\;$$

(3) $$\varepsilon =\frac{\beta }{4\tan\theta }\;$$

**Figure 3:**
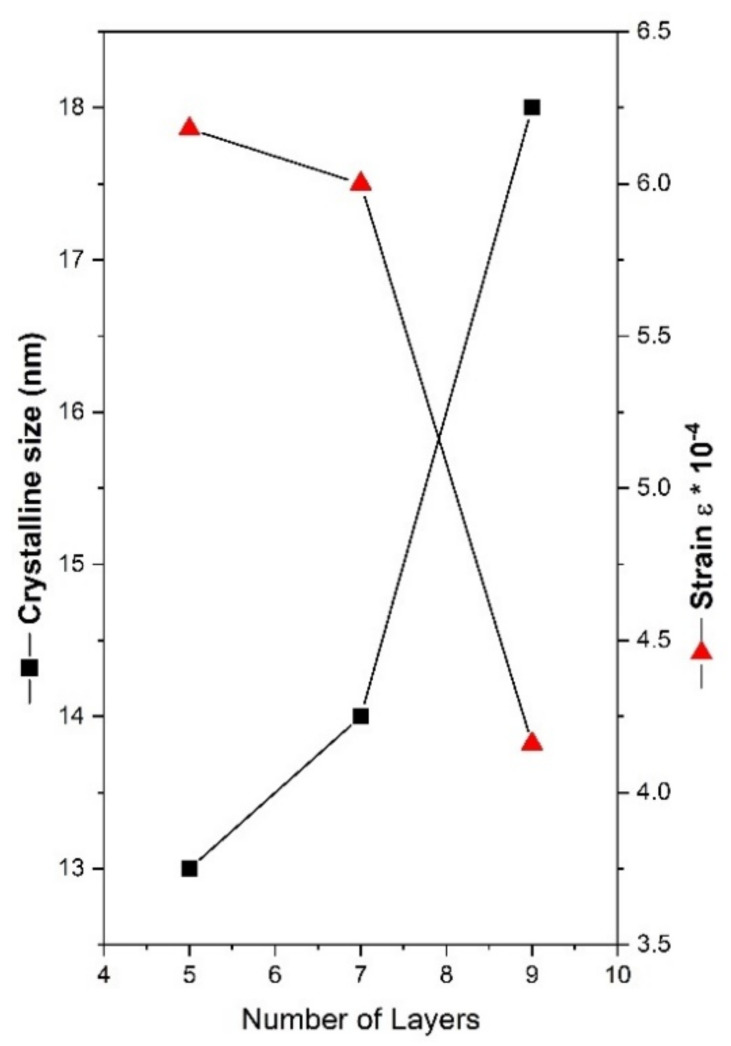
Crystallite size and strain of TiO_2_-SiO_2_ thin films based on the quantity of dip-coating layers.

Figure 3 illustrates how the number of layers of TiO_2_ affected the variance in dislocation density, crystallite size, and strain in the thin films studied. The following values demonstrate that the strain decreases as the number of layers are enhanced and the crystallite size increases. The same trend of the crystallite size was also reported by Lin et al. [22] for ZnO films made with various thicknesses. The relationship between the two variables can be explained by the collective fusing of small crystallite particles into larger ones, which leads to decreased densities of nucleation centers and, in turn, internal strain [23,24].

### 3.2. UV-Vis

**Figure 4:**
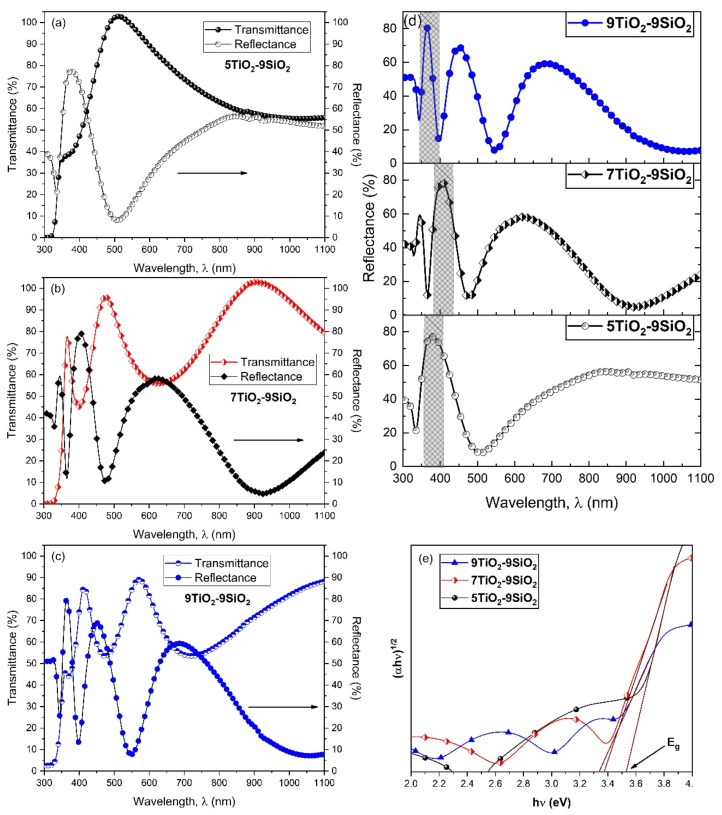
Determination of $E_{g}$ energy for TiO_2_-SiO_2_ thin films. (**a**–**c**) Spectral dependence of T(λ) and R(λ) coefficients, (**d**) UV–Visible reflectance spectra of TiO_2_-SiO_2_ thin films, (**e**) $E_{g}$ determined by optical method using (*α*h*ν*)^1/2^ vs. h*ν* plots of thin films deposited with different numbers of TiO_2_-SiO_2_ layers.

Based on the UV-vis characterization experiments, transmittance (T), and reflectance (R) data on the TiO_2_-SiO_2_ thin films were obtained in the 300–1100 nm wavelength window. Figure 4a–c shows that transmittance spectra are characterized by maxima and minima values of different orders. As the film thickness increases (to 5, 7, and 9 layers), the location of a particular transmittance extrema (maxima) shifts towards longer wavelengths. Similar results have been reported for five TiO_2_ layers on SiO_2_ [25]. Also, the thin films under investigation displayed a dramatic decline in transmittance in the ultraviolet spectrum. It was found that the absorption edge of the transmittance moves towards red in the ultraviolet region as the film thickness grows for TiO_2_ thin films produced with the same withdrawal speed. Figure 4d shows the optical reflectance as a function of wavelength (*λ*). When there is an increase in the number of layers, the reflectance rises, which is more significant within the visible region. The band gap between conduction (CB) and valence bands (VB), a crucial optical characteristic of thin films, is calculated using the well-known Tauc equation, as given in Equation (4) [26]:

(4) $$\alpha h\nu =A{\left(h\nu -E_{g}\right)}^{n}$$

Here, the constant *A* is related to the effective masses (electrons and holes), the input photon energy is represented by h*ν*, the optical bandgap is represented by $E_{g}$, and the exponent n is dependent on the kind of traνnsition. When *n* = 2 denotes permitted indirect transitions, 3 denotes forbidden indirect transitions, 1/2 denotes permissible direct transitions and 3/2 for forbidden direct transitions. When the line is intercepted at the energy axis (α = 0) yields the band gap (Figure 4e). Here, it can be shown that the $E_{g}$ values decrease as the number of TiO_2_ layers increases. The $E_{g}$ value for 9TiO_2_-9SiO_2_ was found to be 3.34 eV, as shown in Figure 4e, which is comparable to observations reported by other groups [27,28,29]. The bandgap energy in Figure 4e for 5TiO_2_ is 3.53 eV, and in this regard, it has been reported that for powders light absorption (as well as the bandgap of TiO_2_), does not change when several layers of TiO_2_ are present. For interacting semiconductor layers, however, it is common to observe a decrease in the band gap due to an increase in the grain size of TiO_2_ layers [30]. Also, some sub-bands form due to defect levels in the forbidden band of TiO_2_, thereby reducing the “band-gap energy” [31].

### 3.3. Raman Spectroscopy

**Figure 5:**
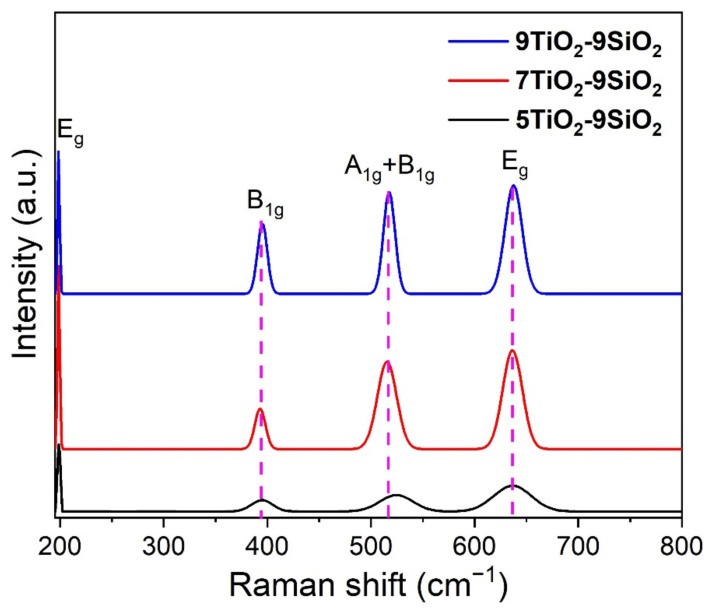
Raman spectrum of TiO_2_-SiO_2_ thin films heated to 550 °C in air (anatase).

Raman measurements of the layers of TiO_2_ deposited on mesoporous SiO_2_ were investigated as described in the experimental section. The resulting Raman data in the 190–800 cm^−1^ range (Figure 5) show that the anatase titania vibrational modes $E_{g}$, B1g, A1g+B1g, and $E_{g}$ have been assigned to peaks at 197, 394, 516, and 637 cm^−1^ in all samples, respectively [32,33]. While the peaks located at 197 and 637 cm^−1^ agree with the $E_{g}$ mode (attributed to the symmetric stretching vibration of O-Ti-O) and a B_1g_ mode corresponds to the peak at 394 cm^−1^ (which is due to the symmetric bending vibration of O-Ti-O), the A_1g_ mode arises from the asymmetric bending vibration of this same bond. The latter mode also overlaps with the remaining B_1g_ mode, producing the emission of a signal with a peak at 516 cm^−1^. This mode was extensively investigated by Otakar et al. [33]. The intensity of the peaks is normally affected by the number of layers [34]. The Raman spectra obtained indicate that the surface of the TiO_2_ thin films is characterized by a pure anatase phase without the production of impurity phases, because there are no rutile (447 and 612 cm^−1^) [35] or brookite (246 and 449 cm^−1^) [36] phase peaks present. As was previously discussed, these results are consistent with XRD data.

### 3.4. Atomic Force Microscopy (AFM)

**Figure 6:**
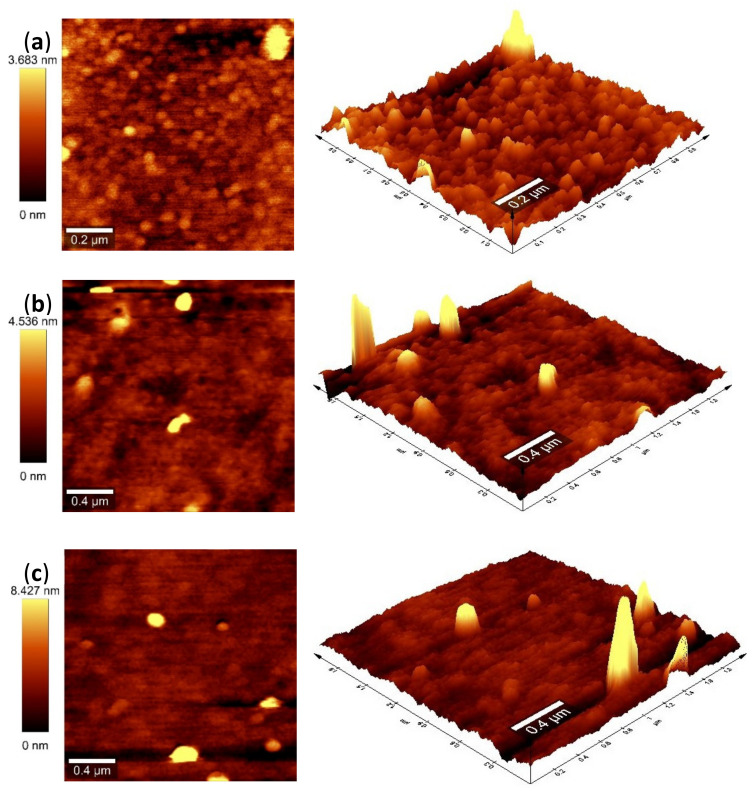
2- and 3-dimensional images of TiO_2_-SiO_2_ thin films with different numbers of layers. (**a**) 5TiO_2_-9SiO_2_ (5 layers), (**b**) 7TiO_2_-9SiO_2_ (7 layers), and (**c**) 9TiO_2_-9SiO_2_ (9 layers).

The TiO_2_-SiO_2_ thin films that were deposited on Corning glass are depicted in Figure 6 in both two- and three-dimensional AFM pictures. The surface morphologies of the films under study reveals porous structures that, interestingly, are characterized by different roughness. Figure 6a shows that 5TiO_2_-9SiO_2_ films are composed by monodisperse spherical particles of a diameter 3.683 nm with a mesoporous structure [37,38]. The main benefit of the reverse micellar route by hydrolysis of titanium isopropoxide to produce TiO_2_ nanoparticles is monodispersity [39]. In this way, the hydration of surfactant polar heads by water molecules is in competition with the hydrolysis process. Growth restrictions and homogeneous particle sizes are caused by the surfactant molecule restructuring that surround the polar species produced during hydrolysis [37]. It is also observed from Figure 6b,c that the 7TiO_2_-9SiO_2_ and 9TiO_2_-9SiO_2_ thin films also have granular microstructures which are composed of ~4.53 nm and ~8.43 nm spheric crystals, respectively. AFM image analysis showed the values of surface roughness in addition to crystal diameter. The root mean square roughness values (R_rms_) of 5TiO_2_-9SiO_2_, 7TiO_2_-9SiO_2_ and 9TiO_2_-9SiO_2_ correspond to 0.508, 0.177 and 0.076 nm, respectively.

### 3.5. Scanning Electron Microscope (SEM)

**Figure 7:**
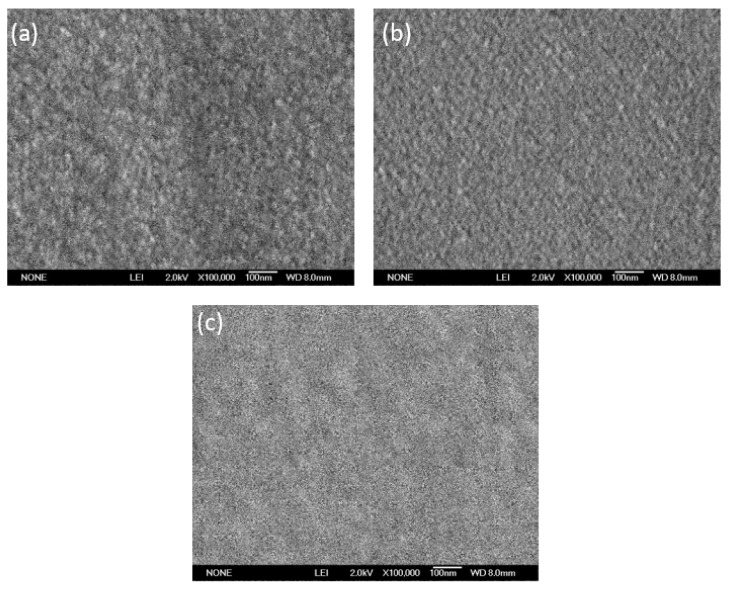
SEM pictures of: (**a**) sample 5TiO_2_-9SiO_2_; (**b**) sample 7TiO_2_-9SiO_2_; (**c**) sample 9TiO_2_-9SiO_2_.

Figure 7 shows the micrographs of thin films. The films under study were annealed at 550 °C; it was observed that the shapes of the formed grains appear to vary according to the number of layers. The most significant differences between the three samples were observed for five layers of TiO_2_ (Figure 7a), seven layers (Figure 7b), and nine layers (Figure 7c). We observed that by increasing the thickness of the TiO2 films, crack formation did not take place, suggesting not only a relatively strong structure, but also that an increase in the film thickness did not lead to detachment of the deposited layers. For the 5TiO_2_-9SiO_2_ thin film (Figure 7a), less closely packed TiO_2_ particles were dispersed, and a sizable particle increase was observed. As the deposition of the layers in the 7TiO_2_-9SiO_2_ sample (Figure 7b) increased, densely packed and uniform nanoscale particles were observed; similar results have been reported in previous studies of TiO_2_/SiO_2_ films [40]. The image of the 9TiO_2_-9SiO_2_ thin film (Figure 7c) shows an extremely smooth surface involving tiny and dense grains positioned on the surface of the film. These results are comparable to those mentioned by Binyu Yu et al. [41].

### 3.6. High Resolution Transmission Electron Spectroscopy (HRTEM)

**Figure 8:**
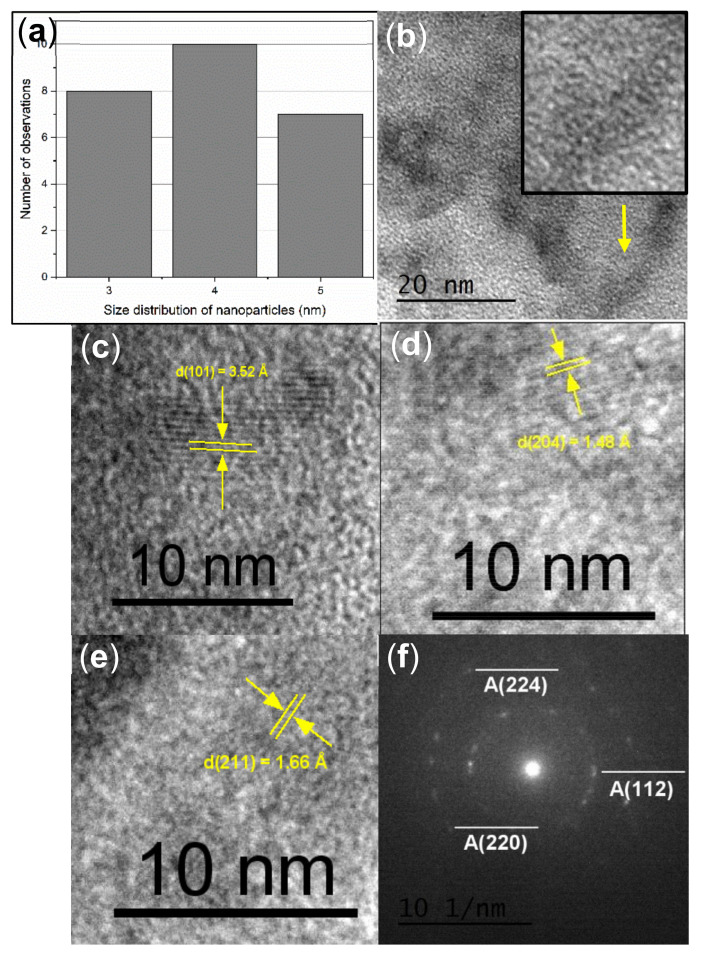
TEM micrography of sample 7TiO_2_-9SiO_2_ (**a**) Size distribution of 7TiO_2_-9SiO_2_ nanoparticles on thin films. (**b**) The disordered wormhole-like pore structure, (**c**–**e**) High-resolution TEM micrograph of 7TiO_2_-9SiO_2_ thin films; crystalline planes are observed. This Figure shows the inter-planar distance obtained from various zones of the sample. (**f**) SAED pattern of 7TiO_2_-9SiO_2_ thin films indexed using anatase TiO_2_ crystallographic parameters.

7TiO_2_-9SiO_2_ thin films were examined using High-resolution TEM (HRTEM) to evaluate their crystalline or amorphous. Size distribution analysis shown in Figure 8a reveals that the average diameter and standard deviation of 7TiO_2_-9SiO_2_ nanoparticles on the surface of the film are about 3–5 nm—a value range that is not consistent with that calculated from XRD experiments. The Scherrer equation, however, is well known to produce a good approximation, and in this regard, the disordered wormhole-like pore structure of the dip-coating prepared sample was confirmed by TEM (see Figure 8b). This material is a good illustration of the type of molecular sieve in which the atomic organization is disordered similarly to amorphous or mesoporous silica, and the channel structure displays a disordered pattern of micropores with a high specific surface area. High-resolution TEM (HRTEM) images of 7TiO_2_-9SiO_2_ in Figure 8c–e revealed the crystalline nature of the nanostructures under study with lattice fringe spacing of 3.52 Å, 1.48 Å, and 1.66 Å, respectively. These can be indexed to (101), (204), and (211) planes of anatase TiO_2_. Figure 8f, on the other hand, shows the transmission electron diffraction pattern of a 7TiO_2_-9SiO_2_ sample. From electron diffraction measurements, the interplanar distances (d) of 7TiO_2_-9SiO_2_ samples were determined to be 2.33 Å, 1.33 Å and 1.16 Å. This result corresponds to anatase phase of TiO_2_. The interplanar distance and the diffraction planes were identified using the powder diffraction files (PDF) #00-021-1272.

### 3.7. Degradation of MB with SiO_2_-TiO_2_ Photocatalyst under UV Irradiation

**Figure 9:**
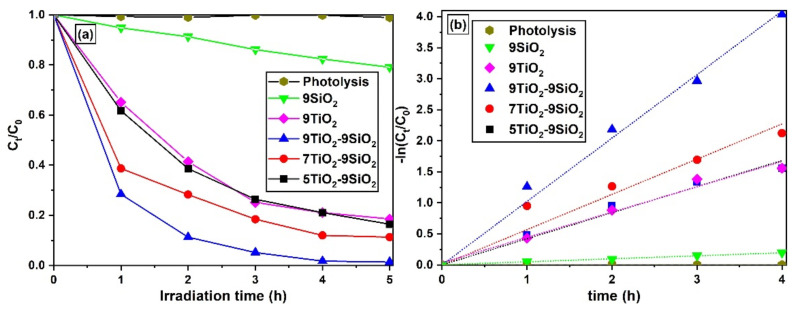
(**a**) Normalized concentration of MB vs UV-light irradiation time in the presence of TiO_2_-SiO_2_ thin films. (**b**) Reaction kinetics of MB photocatalytic degradation at different loadings of TiO_2_-SiO_2_ catalyst.

The main by-products and most worrying pollutants in the textile industry are organic dyes, which are known to degrade when exposed to high levels of photocatalytic activity (PA) in mesoporous TiO_2_. It is particularly important to consider a good excitation during photocatalysis, as the energy of the $E_{g}$ must be exceeded. The TiO_2_ anatase phase has an $E_{g}$ of 3.2 eV, and the corresponding wavelength value of 387 nm showed that light with shorter wavelengths is needed for this project; a wavelength value of 254 nm was selected. TiO_2_ is excited by UV radiation, resulting in valence band holes that oxidize OH^-^ ions or H_2_O to produce the hydroxyl radical (·OH) species. The compound is an extremely potent oxidant that easily oxidizes most organic molecules, converting them to CO_2_, water, and salts [42]. Nevertheless, to improve the absorption efficiency of the TiO_2_, it must be hydrated with some additive, such as P_2_O_5_, carbon, or SiO_2_ [43]. In this work we have deposited SiO_2_ thin films on Corning glass substrates, subsequently, various layers of TiO_2_ were deposited. The opportunity for the hydroxyl radical (·OH) and dye molecules to react during the PA can be enhanced if the thin films absorb more color molecules. The decision was made to utilize the photodegradation of MB to assess the PA of TiO_2_-SiO_2_ thin films for dye discoloration applications. UV/Vis spectroscopy was used to determine the solution’s time-dependent MB concentration. Figure 9a plots the normalized MB concentration under UV radiation as a function of time in the absence of any photocatalyst, in the presence of SiO_2,_ and utilizing various concentrations of thin films. The ratio of C_t_/C_0_ at time t = 0 is calculated as 1, using C_0_ as the initial MB concentration, and C_t_ corresponds to the concentration of unreacted dye at various times. Figure 9a clearly shows that while photolysis does not result in noticeable dye discoloration, MB concentration decreases roughly 20% after 5h for SiO_2_ films, reaching substantially more extensive and faster discoloration performances when using TiO_2_-SiO_2_ films. Since 5TiO_2_-9SiO_2_ materials showed similar activity to that observed for 9TiO_2_ thin films (in which SiO_2_ is not present) and since the photocatalytic activity of TiO_2_-SiO_2_ increases as the number TiO_2_ layers on SiO_2_ is larger, it is possible to suggest that SiO_2_ acts not only as TiO_2_ support in these PA films but as a functional semiconductor layer in the hetero-semiconducting film. As observed from the data in Figure 9a, increasing the number of TiO_2_ layers results in faster discoloration kinetic curves [44,45]. A detailed investigation of the function that particle size plays in pure TiO_2_ photocatalysts under UV light (wavelength 310–330 nm) was made by Zhang et al. They discovered that the kinetics of the electron/hole recombination process depended critically on particle size [44]. According to Afshar et al., the challenges brought on by the drawbacks of pure titanium powder require the manufacture of modified titania. They found that an increased surface area and interaction regions between the SiO_2_ and the titanium nanoparticles could result in a combined impact that can be the primary cause of the high activity of TiO_2_-SiO_2_ [45].

In gas-solid systems, the Langmuir-Hinshelwood (L-H) model has been successfully used to explain the kinetics of heterogeneous catalysis. This model was expanded upon by Al-Ekabi and Serpone [46] to include heterogeneous catalysis of a liquid-solid system. In order to obtain quantitative information on the photocatalytic activity of the as-prepared products, the kinetics of photocatalytic degradation of MB was also investigated. The degradation of MB can be described using the pseudo-first-order Langmuir–Hinshelwood kinetic model as shown below:

(5) $$r=-\frac{dC}{dt}=kC^{n}$$

where *C* is the concentration of the solution, k is the reaction rate constant, and *n* is the order of the reaction. Since photocatalytic oxidation is governed by a first-order reaction, the Equation (6) is obtained:

(6) $$-\frac{dC}{dt}=kC$$

Integrating the Equation (6), and using the following initial conditions, *t* = 0, *C* = *C*_0_, *C*′ = ln *C*_0_, we obtain the Equation (8) in which the reaction rate constant, k, allows for analysis of the efficiency of the dye degradation process.

(7) $$-\ln C=kt+C^{\prime }$$

(8) $$-\ln\left(\frac{C_{t}}{C_{0}}\right)=kt$$

where the apparent first-order rate constant is denoted by k. With increasing TiO_2_-SiO_2_ catalyst loading, it was discovered that the rate constant k increased (Table 1). Multiple heterogeneous photocatalytic systems have used this kinetic model successfully. In Figure 9b, the curves for SiO_2_ and TiO_2_-SiO_2_ with varied layers are presented as −ln(*C_t_*/*C*_0_) vs. *t*. In the presence of TiO_2_, the SiO_2_ thin films photocatalytic activity is enhanced [47]. SiO_2_ thin films have the ability to become acidic, which increases the amount of surface hydroxyl groups [48]. Moreover, surface hydroxyl groups have the ability to take up photoinduced holes from TiO_2_ to produce OH radicals, which can then oxidize absorbed molecules. Hence, Bronsted acidity can be mostly responsible for the increased TiO_2_-SiO_2_ activity, which is consistent with the earlier result by Lu et al. [49]. Simultaneously, it was found that the photocatalytic activity of SiO_2_ could be significantly improved by depositing TiO_2_ thin films on it. As seen in Figure 9b, photocatalysts made of 9TiO_2_-9SiO_2_ are substantially more active than SiO_2_ at degrading MB.

### 3.8. Photocatalytic Mechanism

**Figure 10:**
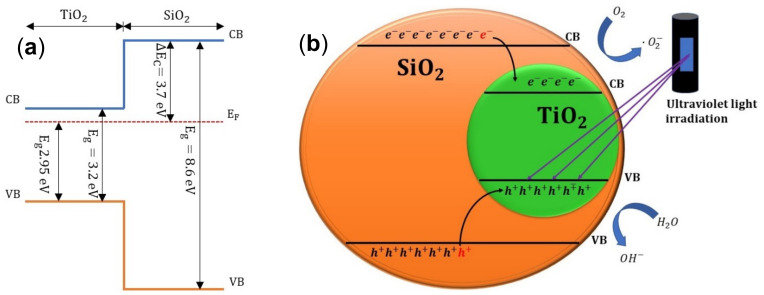
(**a**) TiO_2_-SiO_2_ Band diagram. ∆CB and ∆VB denote the conduction and valence band offset between SiO_2_ and TiO_2_, respectively, and E_F_ denote the Fermi level. (**b**) Photodegradation mechanism of TiO_2_-SiO_2_ under UV light irradiation.

Figure 10a shows a band diagram illustrating the energy levels of the conduction and valence bands for TiO_2_ and SiO_2_. It is important to point out that the conduction band energy of TiO_2_ is lower than that of SiO_2_; therefore, photo-excited electrons on both semiconductor layers are readily transferred to the conduction band of TiO_2_. Inspection of the energy band positions and scheme in Fig 10 also shows that photo-generated holes are promoted to the valence band of TiO_2_ where interphasial oxidation reactions may occur (in Figure 10b this hole-induced oxidation mechanism for methylene blue (MB) is depicted).

In this way, when the photocatalyst absorbs a photon with energy equal to or greater than the band gap energy, the photogenerated electrons and holes are efficiently separated by the space-charge layer, with the holes being transported to the TiO_2_ surface. The primary photon energy-derived elements that interact with H_2_O or OH^-^ adsorbed on the surface to make ·OH are called photogenerated holes [50,51,52]. As can be seen from Figure 10b and the equations that follow:

(9) $${\mathrm{TiO}}_{2}+h\nu \to h^{+}+e^{-}$$

(10) $$h^{+}+H_{2}O\to \cdot \mathrm{OH}+H^{+}$$

(11) $$h^{+}+{\mathrm{OH}}^{-}\to \cdot \mathrm{OH}$$

The superoxide radicals (·O_2_^−^), which are highly oxidant species, are created when the photogenerated electrons interact with the O_2_ on the surface. In fact, these reactions produce a variety of active free radicals with high oxidizing characteristics, such as (·OH, ·O_2_^−^, and HOO·). It should be highlighted, too, that the high rate of photogenerated e^−^ h^+^ recombination makes less effective photodegradation of challenging contaminants. According to [53,54], the interaction between TiO_2_ and SiO_2_ improves the separation of charge carriers (e^−^ and h^+^) and makes it easier for them to transfer between one another. As a result, the suppression of electron-hole recombination is only partially effective, leading to an increase in photoactivity.

## 4. Conclusions

The TiO_2_-SiO_2_ thin films’ structural, optical, and photocatalytic properties are synthesized and evaluated in this work, as well as the significance and importance of TiO_2_ modified SiO_2_ substrates. It has been demonstrated that TiO_2_-SiO_2_ thin films have high photocatalytic performance for the discoloration of Methylene Blue solutions, and likely for many diverse types of organic contaminants in aqueous effluents. The presence of the TiO_2_ layers on top of SiO_2_ thin films improved the photocatalytic properties of the films, due to the photo-generated electrons gathered in the TiO_2_ conduction band and were drawn to the SiO_2_ trap level, which inhibited the recombination of electrons and holes. The presence of nine layers of TiO_2_ in the surface of SiO_2_ yielded superior photocatalytic activity to the other samples. In the end, effective degradation of the model dye was achieved, which is crucial for the detoxification of water. In the future, by further controlling nanocrystals sizes and layers and mesopore sizes precisely, we can expect enhanced photocatalytic properties.

## Figures and Tables

**Table 1:** Results of the photodegradation of MB.

Sample	k (h^−1^)	*R* ^2^	Degradation (%)
9SiO_2_	0.048	0.997	21
9TiO_2_	0.406	0.982	82
5TiO_2_-9SiO_2_	0.397	0.982	83
7TiO_2_-9SiO_2_	0.498	0.956	89
9TiO_2_-9SiO_2_	0.978	0.993	98

## Data Availability

The datasets used and/or analyzed during the current study are available from the corresponding author on reasonable request.
